# Do open youth unemployment and youth programs leave the same mental health scars? - Evidence from a Swedish 27-year cohort study

**DOI:** 10.1186/s12889-015-2496-5

**Published:** 2015-11-20

**Authors:** Mattias Strandh, Karina Nilsson, Madelene Nordlund, Anne Hammarström

**Affiliations:** Department of Social Work, Umeå University, SE-90187 Umeå, Sweden; Centre for Research on Child and Adolescent Mental Health, Karlstad University, SE-651 88 Karlstad, Sweden; Department of Sociology, Umeå University, SE-90187 Umeå, Sweden; Department of Public Health and Clinical Medicine, Umeå University, SE-90187 Umeå, Sweden

**Keywords:** Youth unemployment, Youth programs, Mental health, Life course, Unemployment scarring

## Abstract

**Background:**

Recent findings suggest that the mental health costs of unemployment are related to both short- and long-term mental health scars. The main policy tools for dealing with young people at risk of labor market exclusion are Active Labor Market Policy programs for youths (youth programs). There has been little research on the potential effects of participation in youth programs on mental health and even less on whether participation in such programs alleviates the long-term mental health scarring caused by unemployment. This study compares exposure to open youth unemployment and exposure to youth program participation between ages 18 and 21 in relation to adult internalized mental health immediately after the end of the exposure period at age 21 and two decades later at age 43.

**Methods:**

The study uses a five wave Swedish 27-year prospective cohort study consisting of all graduates from compulsory school in an industrial town in Sweden initiated in 1981. Of the original 1083 participants 94.3 % of those alive were still participating at the 27-year follow up. Exposure to open unemployment and youth programs were measured between ages 18–21. Mental health, indicated through an ordinal level three item composite index of internalized mental health symptoms (IMHS), was measured pre-exposure at age 16 and post exposure at ages 21 and 42.

Ordinal regressions of internalized mental health at ages 21 and 43 were performed using the Polytomous Universal Model (PLUM). Models were controlled for pre-exposure internalized mental health as well as other available confounders.

**Results:**

Results show strong and significant relationships between exposure to open youth unemployment and IMHS at age 21 (OR = 2.48, CI = 1.57–3.60) as well as at age 43 (OR = 1.71, CI = 1.20–2.43). No such significant relationship is observed for exposure to youth programs at age 21 (OR = 0.95, CI = 0.72–1.26) or at age 43 (OR = 1.23, CI = 0.93–1.63).

**Conclusions:**

A considered and consistent active labor market policy directed at youths could potentially reduce the short- and long-term mental health costs of youth unemployment.

**Electronic supplementary material:**

The online version of this article (doi:10.1186/s12889-015-2496-5) contains supplementary material, which is available to authorized users.

## Background

Youth unemployment rates have in many countries been twice as high as those for all adults since the great recession and despite the recovery, the OECD expects that youth unemployment will remain high for some time [1]. Several studies indicate that this may have direct or short term adverse health effects; in particular, unemployment experiences have been linked to poor mental health [2, 3]. In addition, results indicate that youth unemployment may have long-term mental health costs.

Longitudinal studies on adult populations [4–6] and youth panel studies [7–9] have shown that both current unemployment and past unemployment experiences are associated with reduced mental health. These findings are supported by the results of long-term youth cohort studies showing a negative relationship between youth unemployment and subsequent adult mental health. Wadsworth, Montgomery and Bartley identified a relation between unemployment among individuals aged 16–27 and deteriorated mental health at age 33 in a British national birth cohort [10]. Mossakowski detected a similar relationship in the American National Longitudinal Study of Youth, which followed youths over 15 years [11]. Similarly, Swedish studies using the North Sweden Cohort identified negative relationships between youth unemployment at ages 16–21 and mental health at ages 30–42 [12, 13].

The long-term mental health scarring associated with youth unemployment raises questions about the extent to which policies can mitigate such effects. Active labor market policy (ALMP) programs for youth, commonly called youth programs, such as various types of training and activation programs have long been the main policy tool used to help young people at risk of labor market exclusion [14]. Research on youth programs has focused heavily on labor market effects; there has been little emphasis on their potential positive effects on mental health among the young unemployed. Studies that have investigated the relation between ALMPs in general and mental health have yielded very mixed results. In a review of evaluations of short term vocational interventions, Audhoe et al. find only weak support for reductions in mental distress [15]. However, more positive results were obtained in studies examining individual programs [16–18].

The aim of this study is to compare the effects of exposure to open youth unemployment and exposure to participation in youth programs between ages 18 and 21 on adult mental health immediately after the end of the exposure period at age 21 and two decades later at age 43. This is done using the “Northern Swedish Cohort” (NoSCo), a 27- year prospective cohort allowing for the control of pre-exposure mental health, measured at age 16, as well as pre-exposure confounders related either to the probability of exposure to open unemployment and youth programs and/or mental health at age 21 or age 43. The study has a focus on depressive, anxiety, and panic related symptoms. This is conceptualized through using the widely used overarching term ‘Internalized Mental Health Symptoms’ (IMHS).

## Methods

This study uses the “Northern Swedish Cohort” (NoSCo), a prospective cohort study of all pupils in a medium-sized industrial town in northern Sweden (*n* = 1083) who completed or should have completed their final year of compulsory school in 1981 at age 16. This cohort was investigated using a comprehensive questionnaire containing more than 90 questions covering areas such as somatic and mental health, health behavior and labor market experiences. The participants were revisited with the same questionnaire at ages 18, 21, and 30, and most recently at age 43. The initial response rate was 99.7 % and the 27-year follow-up response rate was 94.3 % (*n* = 1010) of those still alive (*n* = 1071). The population is known to be representative of their age cohort on the national level in terms of demography, socioeconomic position, health and health behaviors although the local unemployment levels in their home town were initially higher than the national average [19, 20].

The study, including consent methodology, has ethics approval from the Ethics Committees of Uppsala University, Umeå University and Statistics Sweden as well as by the Regional Ethics Vetting Board in Umeå. Written consent has not been requested from these committees. The respondent is regarded as giving written consent when answering the questionnaire. Participants were/are able to opt out at any time simply by not completing any of the waves of the survey.

### Exposure to open unemployment and youth programs

In order to measure exposure to open unemployment (here defined as actively seeking work while not being in employment, education or training) and participation in youth programs we use a battery of questions from the NoSCo that asked the 21 year-old respondents how many weeks they had spent in employment, studying, in open unemployment, and participating in youth programs since their previous interview at age 18. From these responses we constructed two variables measuring cumulative exposure (in months) to open unemployment and participation in youth programs between ages 18 and 21. In order to exclude frictional exposure (many individuals will for example be registering short periods of unemployment exposure when simply switching jobs) and focus on substantial exposure these variables were then dichotomized into more or less than 6 months of open unemployment between 18 and 21, and more or less than 6 months’ participation in youth programs between 18 and 21 (where less than six months is treated as no exposure). Six months exposure here represent the Swedish administrative definition of long term unemployment for youth. Additionally analyses were also performed, and are presented, using a combination variable of exposure 18–21 (no exposure, open unemployment, youth programs, both youth programs and open unemployment), where the cut off for exposure was lowered to five months in order to ensure a large enough group only exposed to open unemployment (*n* = 39). This is done to test the sensitivity of results to combinations of exposure. Sensitivity analyses further show results to be similar when using exposure to open unemployment and exposure to youth programs measured in number of months.

### The dependent variables

The dependent variables in the study were identical indexes focusing on depressive, anxiety, and panic related symptoms, which can be conceptualized as internalized mental health symptoms (IMHS), at ages 21 and 43. Three questions based on measures of internalized mental health symptoms as defined at the time of the original survey [19, 21] and taken from well-known and validated surveys [22, 23] were used to create a composite index based on a theoretical and clinical evaluation of the seriousness of individual symptoms and combinations of different symptoms [24]. Respondents were asked whether they had or had not (no = 0 and yes = 1) experienced worry/anxiousness and anxiety/panic during the last 12 months. In addition, they were asked how often during the past 12 months they had experienced sadness or felt low on a 4-grade ordinal scale variable coded as never = 0, sometimes = 1, often = 2, always = 3. An ordinal scale index was created based on these questions ranging from 0 = not experiencing any symptoms to 8 = often feeling both worry/anxiousness and anxiety/panic while also often or always feeling sad and low. The order of the values in the scale was based on clinical judgments of the seriousness of these mental health symptoms as well as the frequency of symptoms. Experiencing anxiety/panic was for instance regarded as more serious in as compared to being worried/anxious. A detailed description of the order of the values in the scale is presented in Additional file 1: Table S1.

The mean IMHS score for all ages was low (1.13 at age 16, 1.22 at age 21 and 1.50 at age 43). The distribution of respondents on the index was skewed in a way that could be expected in relation to mental health problems with the most common value being 0 at all ages, which represented about half of the respondents, followed by 1 and so on.

### Confounders

Because previous studies have demonstrated reverse causality between mental health and unemployment [25, 26] it is essential to control for baseline mental health. This was done by using the respondents’ *IMHS at age 16* (which was created in the same way as the dependent measures for ages 21 and 43) as a measure of their mental health before potential exposure to youth unemployment. We also controlled for *semesters in education at ages 18–21* to reflect the fact that not all youths face unemployment risks. Many youths in the cohort spent at least some of the period between ages 18 and 21 in regular education. We controlled for this by creating a variable that counted the number of spring or autumn semesters spent in education during the period of interest. This variable took values of 0–8, with observations evenly distributed over the range. Youths with higher scores had correspondingly lower risks of exposure.

We also controlled for seven pre-exposure confounders relating to youth unemployment and IMHS at 21 and/or 43: *gender*, *long-term somatic health problems* at 16, *truancy* at 16, *parental social class* at 16, *parental employment* at 16, *living with both parents* at 16, and *paternal health problems* at 16. These confounders were chosen among those available on the basis of them having either a relationship with either with the probability of exposure to open unemployment and youth programs and/or IMHS. Respondents were identified as having long-term somatic health problems if they reported suffering from diabetes mellitus, hypothyroidism, neurological disorder, impaired hearing, impaired vision, asthma or eczema. Truancy was scored on a scale ranging from 1 to 5 (1 = truancy at least once a week; 5 = no truancy ever). Three *parental social class* categories based on the parents being manual workers (blue collar workers) or professional workers (white collar workers) were defined: both parents blue collar, one blue collar and one white collar, and both white collar. Two *parental employment* categories were defined: both parents employed and one or both parents unemployed. Respondents were identified as having *paternal health problems* if their father suffered from alcoholism or physical/mental ill health.

### Statistical analysis

The dependent variables, IMHS at ages 21 and 43, were created from a theoretical clinical perspective and can be considered to be functional on an ordinal level. To accommodate this, ordinal regressions were performed using the Polytomous Universal Model (PLUM) in SPSS with the logit link function. This procedure reports odds ratios in a way similar to that used for binary logistic regression models, but the possible outcomes are expanded. The difference is that ordinal regression relates to the odds of one group having a higher or lower score on the dependent variable than another [27]. The regression coefficients are not dependent on the steps of the ordinal dependent variable. Different equations (the number of categories of the ordinal dependent variable −1) are calculated, each with a different intercept, but with the same slopes [28]. Because these intercepts are not used to interpret the results they are not reported in the tables. To assess the results’ robustness, all of the analyses were tested using alternative approaches with different data requirements. The substantive results were not changed by using binary logistic regressions (dichotomizing IMHS as no symptoms = 0, one or more symptoms = 1) or by treating IMHS as a continuous variable (ranging from 0 to 8) and using a repeated measures linear mixed models approach with random intercepts.

To facilitate the interpretation of the odds ratios for the two different types of unemployment exposure in relation to each other and the confounders, the analyses in Tables 2 and 3 are built up stepwise. In both tables, Model 1 includes only exposure to open unemployment together with baseline IMHS at age 16; Model 2 includes only exposure to youth programs together with baseline IMHS at age 16; Model 3 includes exposure to both open unemployment and youth programs together with baseline IMHS at age 16; and Model 4 adds all measured pre-unemployment exposure confounders. No significant interactions between exposure to open unemployment and exposure to youth programs or between the exposure variables and any other variable were identified. Therefore the models do not include interaction terms.

In addition analyses are performed in Table 4 using only one fully controller model for IMHS at age 21 and age 43 respectively. This is done to test the sensitivity of results to combinations of exposure.

All statistical analysis was performed using SPSS v.22.0 (IBM Corp., Armonk, NY, USA).

## Results

As shown in Table 1, youth unemployment was relatively common in our sample despite the very low national adult unemployment levels during this period (adult unemployment was only around 3 % in Sweden during the 1980s). Among the respondents, 14 % had at least 6 months’ exposure to open unemployment while 33.7 % had at least 6 months’ exposure to youth programs between the ages of 18 and 21. The mean cumulative exposures to open unemployment and youth programs were 2.5 and 6.8 months, respectively. Interestingly, many of those who participated in youth programs were not exposed to open unemployment.

**Table 1:** Exposure to open unemployment and youth programs age 18–21, proportion and mean duration

Variables	
Unemployment exposure 18–21 (*n* = 995)	Proportion
Open unemployment for >6 months at 18–21 (*n* = 139)	14.0
Participation in youth programs for > 6 months at 18–21 (*n* = 335)	33.7
	Mean (SD)
Months in open unemployment at 18–21	2.5 (5.9)
Months in youth programs at 18–21	6.8 (11.2)

*SD* standard deviation

The analyses of the relationships between exposure to open youth unemployment and youth programs and IMHS immediately after the end of the exposure are presented in Table 2. Models 1 and 2, which are only adjusted for pre-exposure IMHS, clearly show that exposure to open youth unemployment has a strong and significant relationship with IMHS at 21 (OR = 2.53, CI = 1.82–3.52) but no such relationship exists for exposure to youth programs (OR = 1.24, CI = 0.97–1.59). This does not change when exposure to open unemployment and youth programs are considered together in model 3. The strong and significant relationship between open unemployment and IMHS at 21 persists when the confounders are considered in model 4 (OR = 2.48, CI = 1.57–3.60) but again no such relationship is observed for exposure to youth programs (OR = 0.95, CI = 0.72–1.26).

**Table 2:** Odds ratios from PLUM-ordinal regressions relating IMHS at 21 and exposure to open unemployment or youth programs at 18–21

	Model 1		Model2		Model 3		Model 4^a^	
	OR	95 % CI	OR	95 % CI	OR	95 % CI	OR	95 % CI
Open unemployment 18–21 (no exp. ref.)								
Yes	2.53***	1.82–3.52			2.53***	1.77–3.60	2.48***	1.57–3.60
Youth programs 18–21 (no exp. ref.)								
Yes			1.24	0.97–1.59	1.01	0.78–1.31	0.95	0.72–1.26
IMHS at age 16	1.58***	1.45–1.72	1.58***	1.45–1.72	1.58***	1.45–1.72	1.46***	1.34–1.60
Pseudo R-square								
Cox and Snell	0.12		0.10		0.12		0.15	
Nagelkerke	0.13		0.11		0.13		0.16	

^a^ Covariates added in model 4: Gender, long-term somatic health problems at 16, truancy at 16, parental social class at 16, parental employment at 16, living with both parents at 16, paternal health problems at 16

*** = *p* < 0.001. ** = *p* < 0.01. * = 0.05, OR = Odds Ratio, 95 % CI = 95 % Confidence Interval

Table 3 illustrates the relationship between exposure to open unemployment and youth programs and IMHS at age 43. Model 1, which is only adjusted for pre-exposure IMHS, indicates that exposure to open unemployment has a strong and significant relationship with IMHS at 43 (OR = 1.99, CI = 1.43–2.74). Model 2 indicates that there is a similar but substantially weaker relationship between youth program exposure and IMHS at 43 (OR = 1.41, CI = 1.13–1.65). When analysed together in model 3 the odds ratios for both of exposure drop somewhat, but both remain significantly correlated with IMHS at 43. When all confounders are added in model 4, exposure to open unemployment retains a strong significant relationship with IMHS at 43 (OR = 1.71, CI = 1.20–2.43) but the odds ratio for exposure to youth programs drops and becomes statistically insignificant (OR = 1.23, CI = 0.93–1.63).

**Table 3:** Odds ratios from PLUM-ordinal regressions relating IMHS at 43 and exposure to open unemployment or youth programs at 18–21

	Model 1		Model2		Model 3		Model 4^a^	
	OR	95 % CI	OR	95 % CI	OR	95 % CI	OR	95 % CI
Open unemployment 18–21 (no exp. ref.)								
Yes	1.99***	1.43–2.74			1.77***	1.26–2.48	1.71**	1.20–2.43
Youth programs 18–21 (no exp. ref.)								
Yes			1.41**	1.13–1.65	1.30*	1.01–1.66	1.23	0.93–1.63
IMHS at age 16	1.32***	1.23–1.43	1.32***	1.23–1.43	1.32***	1.23–1.43	1.26***	1.15–1.38
Pseudo R-square								
Cox and Snell	0.06		0.05		0.06		0.09	
Nagelkerke	0.06		0.06		0.07		0.09	

^a^ Covariates added in model 4: Gender, long-term somatic health problems at 16, truancy at 16, parental social class at 16, parental employment at 16, living with both parents at 16, paternal health problems at 16

*** = *p* < 0.001. ** = *p* < 0.01. * = 0.05, OR = Odds Ratio, 95 % CI = 95 % Confidence Interval

Table 4 presents analyses performed using the combination variable of exposure 18–21 (no exposure, open unemployment, youth programs, both youth programs and open unemployment), where the cut off for exposure had to be lowered to 5 months. Only one fully controlled model is presented for IMHS at age 21 and IMHS at age 43 respectively. Looking at IMHS at 21 exposure to open youth unemployment only has a strong and significant relationship (OR = 4.17, CI = 2.29–7.61), no such relationship exists for exposure to youth programs only (OR = 1.19, CI = 0.88–1.59), while exposure to both open youth unemployment and youth programs has a significant but less strong relationship with IMHS at age 21 than open youth unemployment only (OR = 2.31, CI = 1.51–3.49). At age 43 exposure to open youth unemployment only still has a strong and significant relationship with IMHS (OR = 2.29, CI = 1.27–4.14), exposure to youth programs only has a weak but statistically significant relationship with IMHS (OR = 1.35, CI = 1.01–1.85), while exposure to both open youth unemployment and youth programs has a significant relationship with IMHS at age 21 (OR = 2.07, CI = 1.38–3.12).

**Table 4:** Odds ratios from PLUM-ordinal regressions relating IMHS at age 21 and 43 and exposure to different combinations of open unemployment or youth programs 18–21^a^

	IMHS at age 21		IMHS at age 43	
	Model2		Model 2	
	OR	95 % CI	OR	95 % CI
Exposure to unemployment 18–21 (no exp. ref.)				
Exposure to open unemployment 18–21	4.17***	2,29–7.61	2.29**	1.27–4.14
Exposure to youth programs 18–21	1.19	0.88–1.59	1,35*	1.01–1.85
Exposure to both forms 18–21	2.31***	1.51–3.49	2.07***	1.38–3.12
IMHS at age 16	1,49***	1.36–1.63	1.25***	0.15–0.37
Pseudo R-square				
Cox and Snell	0,15		0.09	
Nagelkerke	0.16		0.09	

^a^ Covariates added in the models: Gender, long-term somatic health problems at 16, truancy at 16, parental social class at 16, parental employment at 16, living with both parents at 16, paternal health problems at 16

*** = *p* < 0.001. ** = *p* < 0.01. * = 0.05, OR = Odds Ratio, 95 % CI = 95 % Confidence Interval

Results in Table 4 seem to mainly be in line with what was found in Tables 2 and 3. The main difference appear to be that the significance found for exposure to youth programs at age 43 for the initial models in Table 3 remains (just about) significant when analysed as the group only exposed to youth programs also when all confounders are controlled for. The coefficient is however very low as compared with the two other types of exposure.

## Discussion

The aim of this study was to investigate how exposure to open unemployment and exposure to participation in youth programs between the ages of 18 relate to internalized mental health symptoms at ages 21 and 43. The results showed a relatively strong link between open youth unemployment and both short- and long-term mental health scarring. Exposure to open unemployment between ages 18 and 21was strongly and significantly linked to poorer internalized mental health at age 21 and in middle age at 43. These findings are very similar to what previously have been found in long term youth cohort studies where exposure to youth unemployment has been connected with poorer mental health in middle age [10–13]. An important thing to remember here however is that even though the relationship between open youth unemployment and mental health in adulthood is relatively strong both in this study, as in previous studies cited, open youth unemployment does not explain a great deal of the overall variation in mental health in adulthood. The pseudo R^2^ values of the models in this paper indicate that the majority of variation in IMHS, particularly at age 43, is explained by factors unrelated to open youth unemployment.

The results further showed that exposure to participation in youth programs was not associated with short- or long-term mental health scarring in the same way as exposure to open youth unemployment. Exposure to youth programs at 18–21 was unrelated to internalized mental health at age 21 and only weakly related to internalized mental health at age 43. This relatively clear finding would appear to be contrary to most findings from evaluations of shorter term vocational interventions into unemployment, which find at best weak support for reductions in mental distress in relation to open unemployment [15]. Similarly it deviates from most non-evaluation panels of the relationship between participation in Active Labour Market Policy programs and mental health [29–31], although it would seem to be more in line with the findings from a Swedish panel study of unemployed adults [32] and longitudinal analyses of unemployed adults in the British Household Panel Survey (BHPS) [33], both of which showed improved subjective well-being among unemployed vocational training participants.

There are two major differences between the current study and previous studies of the relationship between participation in Active Labour Market Policy programs and mental health that could help explain the findings in this study. Firstly, previous studies have mainly had a focus on adults. Youth and the entry period into the labor market, which has been in focus for the current article, could here be a particularly sensitive period in the development of young people’s identities and their socialization into the adult world [7, 12]. This could mean that program participation, as compared with open unemployment, could be particularly beneficial for mental health during this period. Secondly, previous studies lack a long-term perspective on the relationship between participation in programs and mental health. In almost all cases, the relationship between the two was only evaluated six months or a year after the end of program participation. Given previous findings of long term mental health scarring of open youth unemployment it could well be the case that beneficial effects of program participation for mental health, as compared with open unemployment, also should be evaluated over longer time periods.

The title of this article asks whether open youth unemployment and youth programs produce similar mental health scars; our results indicate that they do not. Exposure to open youth unemployment appear to be substantially more destructive than exposure to youth programs. Specifically, the former creates both short- and long-term mental health scarring whereas the latter leaves no or minimal scars. Previous research essentially present us with two possible explanations for this difference in the relationship with long term mental health that merit consideration.

Mental health scarring from open youth unemployment could be related to some of the destructive psychological aspects of being in open unemployment. This could for instance be the lack of the psychological functions provided by employment such as time structure, identity, social contacts, regular activity and participation in collective purposes [34]. Youth could here, as suggested above, also be a particularly sensitive period in relation to these factors leaving particularly large mental health scars of exposure to open unemployment. The cognitive activation theory of stress would for instance suggest that unemployment exposure during a sensitive phase in life could lead to diminished long-term coping and experienced hopelessness or learned helplessness [35]. Participation in youth programs could contrary to open youth unemployment, from this perspective, lack some of the destructive psychological aspects of open unemployment. It could for instance fulfil part or all of the young individuals’ suggested need for time structure, identity, social contacts, regular activity and participation in collective purposes. This difference between open youth unemployment and participation in youth programs would then lead to participation in youth programs leaving no or only small mental health scars.

Alternatively, the difference in mental health scarring between open youth unemployment and participation in youth programs could be related to the manifest purpose of youth programs. In the economic literature experiences of open youth unemployment have been found to lead to different forms of socioeconomic scarring, such as increased risk of further unemployment and worse income development [36–38]. This process of socioeconomic scarring could lead to “social chain reactions” [39] whereby the initial open youth unemployment experience leads to a non-optimal socioeconomic career and exposure to conditions that are not conducive to good mental health, thus leading to the found long term mental health scars of open youth unemployment. Youth programs are policy tools that on a manifest level aim to help young people at risk of labour market exclusion through activities such as activation, education, and training. This is done primarily to enhance the young individual’s human capital and through this her/his short and long term prospects on the labour market [14]. If youth programs actually succeed in doing this they should lead to substantially less socioeconomic scarring, as compared with open youth unemployment, and through this produce less mental health scarring connected to “social chain reactions”.

Both of these proposed mechanisms appear to be feasible explanations both for the found mental health scarring of open youth unemployment as well as the limited mental health scarring of participation in youth programs. It is however outside the scope of this article to investigate which of these two, or if it is both, of the proposed mechanisms that explain the found results. We however believe that it is an important and promising task for future research to disentangle the mechanisms behind the found results.

### Strengths and limitations

The main strengths of this study are the very long follow-up time, the availability of information on pre-exposure mental health, and the extremely low attrition rate. The “Northern Swedish Cohort” offers through its 27-year follow up unique opportunities for investigating long term issues such as mental health scarring of unemployment. The availability of pre-exposure mental health that this provides us with is very important in order to limit the risk of results being affected by reverse causality, a factor which always have to be considered when investigating the relationship between unemployment and mental health. Finally, the exceptionally low attrition rate that NoSCo offer over the long time period lower the risk of heterogeneous out selection from the study affecting results. This is particularly important when studying weak groups such as the unemployed, where attrition is a common problem.

The geographical basis of the sample, a medium-sized industrial town in the north of Sweden, is one possible limitation. Although the studied population is generally representative of the equivalent national cohort, the local labor market context and the local availability of youth programs may have affected the results. It is possible that the somewhat higher unemployment rates in the studied town and/or the quality of the local youth ALMP programs may have influenced the results. It is today impossible to judge if this indeed was the case, although it would appear unlikely. If it was the case our results would, however, at least indicate the potential of youth programs in mitigating mental health scarring due to unemployment. A second limitation is the outcome variable, IMHS, which was based on measures of internalized mental health symptoms as defined at the time of the original survey and is functional only on an ordinal scale level. It would here have been better to have properly developed and validated psychological scales available and we would strongly like to encourage research on this theme using such scales. The results do however appear to be robust both in relation to alternative statistical approaches as well as to analyses of the individual items. A third possible limitation is that the number of respondents limited the ability to analyse combinations of exposure in a detailed way. Results with the somewhat less strict combination variable however appeared to support the conclusions although further analyses with larger groups would both be very interesting and desirable.

## Conclusions

We believe that these results have important implications for dealing with Europe’s current high levels of youth unemployment. A considered and consistent active labor market policy directed at youths could potentially reduce the short- and long-term mental health costs of youth unemployment. This would be beneficial in terms of individual mental health and also for the long-term prospects of European youths once the labor market recovers.

## Additional file

Additional file 1: Table S1. The internalized mental health symptoms index (range 0 - 8)*. The table shows which combinations of the three variables that comprise the index correspond to which index values. (DOCX 14 kb)
