# Supplementary material for: Do open youth unemployment and youth programs leave the same mental health scars? - Evidence from a Swedish 27-year cohort study
Source: BMC Public Health. 2015 Nov 20;15:1151. doi: 10.1186/s12889-015-2496-5 (PMC4654869; doi:10.1186/s12889-015-2496-5)
Supplement: Additional file 1: Table S1. — The internalized mental health symptoms index (range 0 - 8)*. The table shows which combinations of the three variables that comprise the index correspond to which index values. (DOCX 14 kb) [file 12889_2015_2496_MOESM1_ESM.docx]

**Additional file 1**

**Table S1. The Internalized Mental Health Symptoms index (range 0 - 8)*. The table shows which combinations of the three variables that comprise the index correspond to which index values.**

| **Index** | **Worry/anxiousness** |  | **Anxiety/Panic** |  | **Frequency of feeling sad/low** |
| --- | --- | --- | --- | --- | --- |
| **0** | no - worry/anxiousness | **and** | no - anxiety/panic | **and** | never felt sad/low |
| **1** | no - worry/anxiousness | **and** | no - anxiety/panic | **and** | felt sad/low sometimes |
| **2** | yes - worry/anxiousness | **and** | no - anxiety/panic | **and** | never felt sad/low |
| **2** | yes - worry/anxiousness | **and** | no - anxiety/panic | **and** | felt sad/low sometimes |
| **3** | no - worry/anxiousness | **and** | yes - anxiety/panic | **and** | not felt sad/low |
| **3** | no - worry/anxiousness | **and** | no - anxiety/panic | **and** | felt sad/low often |
| **3** | no - worry/anxiousness | **and** | no - anxiety/panic | **and** | felt sad/low always |
| **4** | yes - worry/anxiousness | **and** | no - anxiety/panic | **and** | felt sad/low often |
| **4** | yes - worry/anxiousness | **and** | no - anxiety/panic | **and** | felt sad/low always |
| **5** | no - worry/anxiousness | **and** | yes - anxiety/panic | **and** | felt sad/low sometimes |
| **5** | yes - worry/anxiousness | **and** | yes - anxiety/panic | **and** | never felt sad/low |
| **6** | no - worry/anxiousness | **and** | yes - anxiety/panic | **and** | felt sad/low often |
| **6** | no - worry/anxiousness | **and** | yes - anxiety/panic | **and** | felt sad/low always |
| **7** | yes - worry/anxiousness | **and** | yes - anxiety/panic | **and** | felt sad/low sometimes |
| **8** | yes - worry/anxiousness | **and** | yes - anxiety/panic | **and** | felt sad/low often |
| **8** | yes - worry/anxiousness | **and** | yes - anxiety/panic | **and** | felt sad/low always |

*Table adapted from Winefield et al., 2013
